# Laryngeal plasmacytoma in a patient with Down’s syndrome

**DOI:** 10.4322/acr.2024.508

**Published:** 2024-08-02

**Authors:** Hassan Hodroj, Zahraa Saker, Zahraa Al Najjar, Hassane Choukr, Mohamad Reda Noureddine El Moussaoui

**Affiliations:** 1 Lebanese University, Faculty of Medical Sciences, Otolaryngology-Head and Neck Surgery Department, Beirut, Lebanon; 2 Al-Rassoul Al-Aazam Hospital, Research Department, Beirut, Lebanon; 3 Lebanese University, Doctoral School of Science and Technology, Beirut, Lebanon; 4 Al-Rassoul Al-Aazam Hospital, Pathology Department, Beirut, Lebanon; 5 Al-Rassoul Al-Aazam Hospital, Otolaryngology-Head and Neck Surgery Department, Beirut, Lebanon

**Keywords:** Extramedullary plasmacytoma, laryngeal neoplasms, larynx

## Abstract

Extramedullary plasmacytoma is a rare localized plasma cell neoplasm typically found in soft tissues outside the bone marrow. Predominantly occurring in the head and neck region, particularly in the sinonasal and nasopharyngeal areas, it presents a diagnostic challenge due to its uncommon nature. Herein, we report a 38-year-old female patient with Down’s syndrome with a 2-year complaint of intermittent dysphonia, hoarseness, and progressive respiratory distress, including dyspnea, fatigue, and biphasic stridor. Examination via flexible laryngoscopy revealed a white lesion, prompting direct microscopic laryngeal surgery to excise a 1x1 cm mass. Histological findings confirmed the diagnosis as solitary extramedullary plasmacytoma. Notably, this represented the first documented case of laryngeal solitary extramedullary plasmacytoma in a patient with Down’s syndrome. This case underscores the importance of considering tumor development in the larynx among individuals with Down’s syndrome, highlighting the necessity for tailored management strategies to address such occurrences effectively. Increasing awareness of this association can aid in early detection and appropriate treatment of tumors in this population.

## INTRODUCTION

Plasmacytoma is a rare type of cancer that arises from neoplastic monoclonal plasma cells. It is considered an intermediate malignancy between multiple myeloma and gammopathy of undetermined significance.^1^ Plasmacytoma is divided into two groups: solitary plasmacytoma of bone (SPB) and extramedullary plasmacytoma (EMP).^2^

EMP is a rare plasma cell neoplasm usually located in the head and neck, more commonly in the upper aerodigestive tract, and progression to myeloma is uncommon.^3^ It was first reported in 1905, and since then, numerous articles have discussed this rare tumor.^4^ EMP accounts for less than 1% of all the head and neck tumors, up to 0.45% of the malignant laryngeal tumors,^5^ and less than 4% of all plasma cell malignancies.^6^ The median age of EMP patients is 60 years, and two-thirds are males,^5^ with a low incidence of 0.04 cases per 100,000 patients worldwide.^7^ Patients mainly complain of progressive hoarseness, stridor, dysphagia, and cough.^8^ The diagnosis of EMP is mainly pathological, based on the presence of neoplastic monoclonal plasma cells. Radiological examination is also an important diagnostic method that usually reveals a homogenous laryngeal mass with well-defined margins.^9^ Despite the good response to radiotherapy, tumor recurrence occurs in 14-20% of EMP patients.^10^

A unique pattern of malignancy has been exhibited in Down’s syndrome patients.^11^ They have an increased risk of developing acute lymphoblastic leukemia but a decreased incidence of solid tumors compared to the general population,^11^ potentially due to alteration(s) in the immune system and elevated levels of cytokines and growth factors.^12^ Early diagnosis and treatment are clinically critical for Down’s syndrome patients due to the particular profile of treatment-related toxicities.^12^ Previous studies have reported numerous laryngeal diseases in Down’s syndrome patients, such as subglottic stenosis, laryngomalacia, and vocal cord paralysis.^13^ Given the rarity of this neoplasm, we report the first case of laryngeal EMP in association with Down’s syndrome, taking into consideration its clinical and pathological presentation.

## CASE REPORT

A 38-year-old female patient with Down’s syndrome presented to our otolaryngology clinic due to intermittent dysphonia and hoarseness that started 2 years ago with progressive worsening associated with dyspnea, fatigue, and biphasic stridor. Flexible laryngoscopy showed an anterior supraglottic white mass obstructing about 25% of the laryngeal inlet. The vocal cords were mobile with normal glottic closure. The patient underwent microlaryngeal surgery for the mass excision. The lesion was removed with negative margins.

Direct suspension laryngoscopy was performed under general anesthesia using a binocular microscope with a 400 mm objective lens. The mass was white and well-vascularized, extending from the anterior commissure to the infra-supraglottic region of the larynx. The radical-resected soft tissue lesion was 1x1 cm without any significant infiltrate. Systemic antibiotics, non-steroidal anti-inflammatory drugs (NSAIDs), and proton pump inhibitors (PPIs) were administered to prevent the formation of excessive fibrin and granulation tissue and further stenosis.

**Figure 1:**
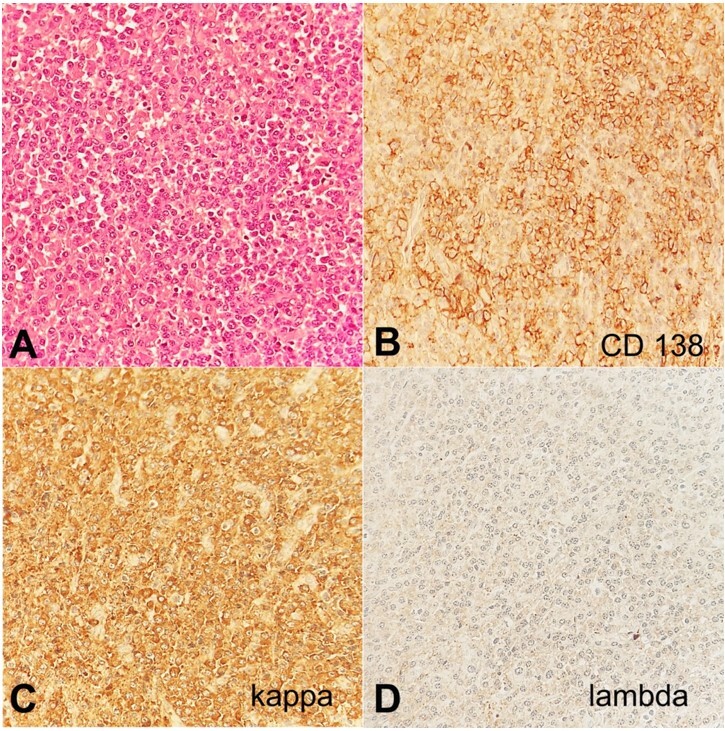
Photomicrograph of the surgical specimen. **A –** abundant proliferated neoplastic plasma cells with basophilic cytoplasm, large nuclei with remarkable nucleoli (H&E, 400X); **B –** expression of prototypical CD138 marker(400X); **C –** high expression of Kappa light chain (400X); **D –** negative expression of the Lambda light chain (400X).

Histological examination showed proliferation of plasma cells in a nodular arrangement. Most neoplastic cells were poorly to moderately differentiated, with large nuclei and basophilic cytoplasm. Immunohistochemical staining revealed positive staining of CD138 and Kappa light chain in the atypical cells but negative staining for Lambda light chain, suggesting the diagnosis of solitary EMP (Figure 1).

Screening for multiple myeloma with whole-body scintigraphy, serum quantitative immunoglobulins, beta-2 microglobulin, serum protein electrophoresis, urine protein electrophoresis, serum protein immunofixation assay, urine protein immunofixation assay, and the ratio of free light chains were all within the normal limits (Table 1). A bone marrow biopsy revealed a 2% invasion of neoplastic plasma cells. The diagnosis of multiple myeloma was excluded. A positron emission tomography (PET) scan of the whole body excluded any other active lesions. The patient was ultimately diagnosed with solitary EMP and was later followed up by an oncologist. The patient kept up without any recurrence or disease progression.

**Table 1:** Laboratory tests for the diagnosis of extramedullary plasmacytoma

**Test**	**Result**	**Normal range**
**Serum immunoglobulins**		
Serum IgA	329	70.0-400.0 mg/dL
Serum IgM	88	40.0-230.0 mg/dL
Serum IgG	1290	700.0-1600.0 mg/dL
**Serum Beta-2 microglobulin**	1.90	0.8-2.34 mg/L
**Serum protein electrophoresis**		
Albumin	53.4	55.8-66.1%
Alpha 1	5.9	2.9-4.9%
Alpha 2	9.7	7.1-11.8%
Beta 1	6.2	4.7-7.2%
Beta 2	5.7	3.2-6.5%
Gamma	19.1	11.1-18.8%
**Urine protein electrophoresis**	Normal profile	-
Monoclonal human antisera		
Anti-Kappa light chain		
Anti-Lambda light chain		
Anti-free Kappa light chain		
Anti-free Lambda light chain		
**Serum protein immunofixation assay**	Absence of monoclonal bands	-
Anti-IgA		
Anti-IgM		
Anti-IgG		
Anti-Kappa light chain		
Anti-Lambda light chain		
**Urine protein immunofixation assay**	Absence of Bence Jones Proteinuria	-
Monoclonal human antisera		
Anti-Kappa light chain		
Anti-Lambda light chain		
Anti-free Kappa light chain		
Anti-free Lambda light chain		
**Serum-free light chains**		
Serum-free Kappa chain	34.8	3.3-19.4 mg/L
Serum-free Lambda chain	27.55	5.71-26.3 mg/L
Free Kapp/Free Lambda ratio	1.26	0.26-1.65

## DISCUSSION

Plasmacytoma is a rare form of cancer derived from neoplastic monoclonal plasma cells, distinguished by localized proliferation. Among plasmacytomas, the prevalence of SBP is approximately 40% higher than EMP occurrence.^2,6^ The etiology of EMP remains unknown, yet it may be related to viral infections or chronic stimulation of inhaled irritants.^2^ SBP usually arises in the spinal column and skulls, whereas EMP is mainly in the nasopharyngeal area.^2^ The average age of diagnosis is between the fifth and sixth decades of life with male predominance, yet it might be diagnosed in the second and third decades of life.^14^ We reported this neoplasm in a Down’s syndrome patient younger than 40 years old.

Most of the EMP lesions occur in the mucosa of the upper respiratory tract, mostly in the nasal cavities and paranasal sinuses, followed by the nasopharynx, tonsils, and oropharynx.^15^ It rarely occurs in the maxillary region.^14^ Yet, our case presented solitary EMP as a supraglottic mass. The most common symptom of laryngeal plasmacytoma is progressive hoarseness, as well as dysphagia, dyspnea, stridor, and cough, taking into consideration that these symptoms can last from months to years before diagnosis.^10^ In this case, the diagnosis was established after two years of progressive worsening of dysphonia and hoarseness over two years in association with dyspnea and biphasic stridor.

The diagnosis of EMP is challenging because it is clinically similar to chronic inflammation diseases, lymphomas, and plasma cell gingivitis.^14^ Magnetic resonance imaging and computed tomography are useful for diagnosing laryngeal plasmacytoma, where the plasmacytoma lesion usually appears as a homogenous, well-defined mass with slight-to-moderate contrast enhancement.^8,9^ As there is no well-defined radiologic diagnostic criterion for EMP, the histopathological view is the gold standard for differentiating EMP from other lesions such as SBP, lymphoplasmacytic lymphoma, and multiple myeloma.^4^ The neoplastic plasma cells typically express the marker CD138, which is indicative of plasma cell lineage. At the same time, the determination of clonality is based on the expression of Kappa and Lambda light chains.^2,4^ The diagnosis of EMP is further accomplished by the expression of a monoclonal light chain, predominantly IgG Kappa chain, which differentiates EMP from the solitary lesion.^15^ Other diagnostic criteria for EMP should be fulfilled to exclude other types of cancers, such as bone marrow biopsy with less than 10% of atypical plasma cells, absence of bone lysis, normal serum or urine proteins electrophoresis, and lack of anemia, hypercalcemia, or renal failure.^2,15^

The rarity and lenghty course of EMP renders early intervention and treatment. Management of EMP usually involves surgical resection when easily accessible, which allows rapid elimination of the symptoms. Adjuvant chemotherapy may be used to prevent further disease progression to multiple myeloma.^5,16^ Appropriate radiotherapy usually results in long-term stability and potential healing in 65% of the patients.^17^ Chemotherapy is not usually recommended, yet it is considered after radiotherapy in large tumors^18^ and/or persistent tumors or recurrences.^9^ Long-term care is vital because EMP may develop into multiple myeloma or other disseminated tumors.^19^

## CONCLUSION

EMP is a rare and aggressive tumor that typically arises in the nasal cavity and paranasal sinuses. This case report highlights the importance of the diagnosis of EMP in Downs' syndrome as an association between certain medical conditions and rare tumors like EMP. While this case report may represent a unique instance of EMP occurring in Down's syndrome, it raises important considerations for further research and clinical practice. Before initiating treatment, a thorough examination of laryngeal masses is crucial for accurate diagnosis and appropriate therapeutic management. Long-term follow-up is imperative as recurrence of EMP can occur even after successful primary therapy, underscoring the need for vigilant monitoring and timely intervention.
